# Prevalence and associated factors of herbal medicine use among pregnant women on antenatal care follow-up at University of Gondar referral and teaching hospital, Ethiopia: a cross-sectional study

**DOI:** 10.1186/s12906-017-1608-4

**Published:** 2017-02-01

**Authors:** Abebe Basazn Mekuria, Daniel Asfaw Erku, Begashaw Melaku Gebresillassie, Eshetie Melese Birru, Balem Tizazu, Alima Ahmedin

**Affiliations:** 1 Department of Pharmacology, College of Medicine and Health Sciences, University of Gondar, Chechela Street, Lideta Subcity Kebele 16, Gondar, Ethiopia; 2 Department of Pharmacy Practice, School of Pharmacy, University of Gondar, Gondar, Ethiopia; 3 Department of Pharmacognosy, School of Pharmacy, University of Gondar, Gondar, Ethiopia; 4 Department of Pharmacology, School of Pharmacy, University of Gondar, P.O. Box: 196, Gondar, Ethiopia

**Keywords:** Pregnant women, Herbal medicine, Maternal health, Ethiopia

## Abstract

**Background:**

Improving maternal and child health is one of the public health priorities in several African countries including Ethiopia. However, research on herbal medicine use during pregnancy is scarce in Ethiopia. The present study aimed at assessing the prevalence and correlates of herbal medicine use among pregnant women on antenatal care (ANC) follow-up at Gondar university referral hospital, Ethiopia

**Methods:**

An institutional-based cross sectional study was conducted on 364 pregnant women attending ANC clinic from March to May 2016 at University of Gondar referral and teaching hospital, northwest Ethiopia. Data on socio-demography, pregnancy related information as well as herbal medicine use was collected through an interviewer-administered questionnaire. Descriptive statistics, univariate and multivariate logistic regression analysis were performed to determine prevalence and associated factors of herbal medicine use.

**Results:**

From 364 respondents, 48.6% used herbal medicine during current pregnancy. ginger (40.7%) and garlic (19%) were the two most commonly used herbs in pregnancy. Common cold (66%) and inflammation (31.6%) were the most common reasons for herbal use. Majority of herbal medicine users (89.8%) had not consulted their doctors about their herbal medicine use. Rural residency (Adjusted odds ratio (AOR): 3.15, Confidence interval (CI): 1.17–6.14), illiteracy (AOR: 4.05, CI: 2.47–6.62) and average monthly income less than 100 USD (AOR: 3.08CI: 1.221–7.77) were found to be strong predictors of herbal medicine use.

**Conclusions:**

The use of herbal medicine during pregnancy is a common practice and associated with residency, level of education and average monthly income. From the stand point of high prevalence and low disclosure rate, the health care providers should often consult pregnant women regarding herbal medicine use.

**Electronic supplementary material:**

The online version of this article (doi:10.1186/s12906-017-1608-4) contains supplementary material, which is available to authorized users.

## Background

Traditional medicine, according to World Health Organization (WHO), is defined as “health practices, approaches, knowledge and beliefs incorporating plant, animal and mineral based medicines, spiritual therapies, manual techniques and exercises, applied singularly or in combination to treat, diagnose and prevent illnesses and maintain well-being” [1]. Among the traditional medicine practices, the use of herbal medicines which is defined as plant-derived preparations claimed to have therapeutic benefits, is the most popular and used by the general population as well as pregnant women around the globe [2–4].

Pregnant women use herbal medicines for different purposes including pregnancy related ailments such as nausea and vomiting, enhancing labor [5] or pregnancy unrelated purposes and ailments such as cold, respiratory illnesses and skin problems as well as nutritional benefit [6]. Furthermore, pregnant women use herbal medicines due to its easy accessibility, assumed better efficacy compared to the modern medicine, traditional and cultural belief in herbal medicines to cure illnesses and comparatively low cost of herbal medicines [7, 8]. The most popular herbal remedies used by pregnant women in the world include ginger (*Zingiber officinale),* garlic (*Allium sativum),* green tea (*Camellia sinensis),* peppermint (*Mentha piperita)* and fenugreek (*Trigonella foenum-graecum,*) [9]. Studies conducted in Australia and Kenya have reported that factors associated with increased use of herbal medicines among pregnant women includes being older and married, low economic status, being less educated and severity of nausea and vomiting [10, 11]. Despite the increased consumption of herbal medicines among pregnant women all over the globe, majority of them are unaware of the potential side effects and a potential teratogenicity of some herbal products [11, 12]. Pregnant and lactating women are especially prone to adverse effects from herbal medicines as the safety profiles and appropriate dosages of most of herbal medicines are not well studied in this group of populations [13].

In Ethiopia, more than 80% of the population use traditional medicine. A study done in Nekemte Hospital, Western Ethiopia showed that 69.8% of pregnant women use herbal medicines and the most common herbs used were ginger (44.4%), garlic (37.3%), and eucalyptus (9.1%) [14]. Traditional medicine including the use of herbal medicines in Ethiopia is not only common but also culturally acknowledged. Yet there is scarcity of data on the prevalence and correlates of herbal medicine use among pregnant women. The present study aimed, therefore, to investigate the prevalence and correlates of herbal medicine use among pregnant women on ANC follow-up at Gondar university referral hospital, Ethiopia

## Methods

### Study design and setting

An institutional-based cross sectional study was conducted on 364 pregnant women attending ANC clinic from March 1 to May 28, 2016 at University of Gondar Referral and Teaching Hospital (UOGRTH), northwest Ethiopia. UOGRTH is located in Gondar town, northwest Ethiopia, 738 km away from Addis Ababa (the capital city of Ethiopia). The hospital is the only referral center in the area with multiple specialized clinics including ANC follow up clinic. Annually, more than 15,000 pregnant mothers visit the hospital for ANC services.

### Sample size determination and procedure

Our source populations were all pregnant women attending ANC clinic of UOGRTH, while those pregnant women who visit the ANC clinic during the data collection period were taken as a study population. Single population proportion formula was used with the assumption of 95% confidence interval, 5% margin of error and 61.7% prevalence [14, 15] of herbal medicine use among pregnant women and 5% for possible non-response was taken to determine a final sample size of 410. According to GURH Statistics and Information Office record, an estimated number of 25 pregnant women visit the ANC clinic every day for ANC services. Thus, the total number of pregnant women who will visit the ANC clinic during the two month study period was calculated. The number of pregnant women to be interviewed per day during the 60 days of data collection was estimated to be 7. By dividing the daily pregnant women visit with the number of pregnant women to be surveyed per day, every fourth pregnant women available at the ANC clinic during the two month data collection period were included by using systematic random sampling technique.

### Data collection method and survey instrument

Data collection was performed by three graduating class pharmacy students through interviewer-administered questionnaire. The investigators were properly trained on the instrument and ways of approaching the patients and securing their permission for interview prior to the data collection process. The data collection tool was developed after a thorough literature review of the published studies [5, 6, 8–11] and prepared in English This was translated to local language (Amharic) and then back to English in order to ensure that the translated version gives the proper meaning. The data collection instrument was pretested on 20 pregnant women who were not included in the final analysis and relevant modifications were done before the commencement of actual data collection. The final questionnaire was constituting 21 items that were divided into two main parts (Annex). The first section was focusing on the socio-demographic and pregnancy related information including age, marital status, educational level, history of ANC visit, current pregnancy status and duration of pregnancy. The second section aimed at assessing the level of herbal medicine use, information source about herbal medicine and discussion with physicians about herbal medicine use. The use of herbal medicine among respondents was assessed by a series of questions including use of herbal medicines during pregnancy, type of herbal medicine used, purpose of use, source of information and any untoward effects encountered while using herbal medicines. Respondents were labeled as herbal medicine users if they have taken herbal medicine(s) via any route of administration during gestational period. Routine meal preparations and those that are taken as nutrients (vitamin supplements) were excluded. The data collection tool along with a cover later is provided in Additional file 1.

### Statistical analysis

The final data collection tool was checked for completeness, and responses were entered into and analyzed using the Statistical Package for the Social Sciences (SPSS) software version 21.0 for Windows. Frequencies and percentages were used to express different variables. Univariate analysis and multivariate logistic regression analysis were used to determine factors associated with herbal medicine use. The results were adjusted for patients’ demographic and pregnancy related characteristics. Odds ratio (OR) with 95% confidence interval (95% CI) were also computed along with corresponding *p*-value (*p* < 0.05) as cut off points for determining statistical significance of associations among different variables.

## Results

### Socio-demographic characteristics

Out of 410 pregnant women invited to participate, 364 of them completed the survey giving a response rate of 88.8%. The mean age of respondents was 26 years with a standard deviation of ±5.0. Majority of the respondents were Orthodox Christians (87.6%) and urban residents (79.7%). About two-third (66.2%) of the subjects drawn in this study were in their third trimester of pregnancy. The socio-demographic and pregnancy related characteristics of respondents are summarized in Table 1.

**Table 1:** Socio demographic characteristics and factors associated with herbal medicine use among respondents, Gondar, 2016 (*N* = 364)

Variables	Herbal medicine use			
	Yes (n)	No (n)	COR (95%CI)	AOR (95% CI)
Age group, in years				
< 20	29	20	0.95 (0.46–1.34)	-
21–30	122	116	0.33 (0.17–1.98)	-
> 30	26	51	1	-
Residence				
Rural	48	26	2.45 (1.80–5.06)	3.15 (1.17–6.14)
Urban	129	161	1	1
Educational status				
Illiterate	41	21	3.14 (1.94–5.78)	4.05 (2.48–6.62)
Primary	40	33	1.70 (1.88–3.30)	2.13 (1.87–5.23)
Secondary	65	60	1.19 (1.03–2.17)	1.99 (0.40–2.22)
Tertiary	31	73	1	1
Religion				-
Orthodox Christian	154	165	1	-
Muslim	19	20	0.91 (0.85–0.97)	-
Others^a^	4	2	1.01 (0.7–1.13)	-
Employment status				
Government employed	45	52	0.83 (0.42–1.64)	0.57 (0.24–1.34)
Self-employed	19	34	0.54 (0.25–1.18)	0.39 (0.16–0.95)
Unemployed	113	101	1	1
Average monthly family income				
< 100	133	105	2.09 (0.96–4.56)	3.08 (1.22–7.77)
100–150	35	38	2.14 (1.064–4.30)	2.60 (1.20–5.63)
> 150	19	34	1	1
Presence of health problem				
No	47	165	0.65 (0.36–1.18)	0.56 (0.29–1.08)
Yes	130	22	1	1
Parity				-
1–2 children	141	131	0.69 (0.31–1.53)	-
3–4 children	27	49	0.89 (0.41–1.93)	-
> 4 children	9	7	1	-

Abbreviations: *AOR* Adjusted odds ratio; COR: Crude odds ratio

^a^Protestant, Jehovah witness

### Prevalence and indication of herbal medicine use

**Fig. 1:**
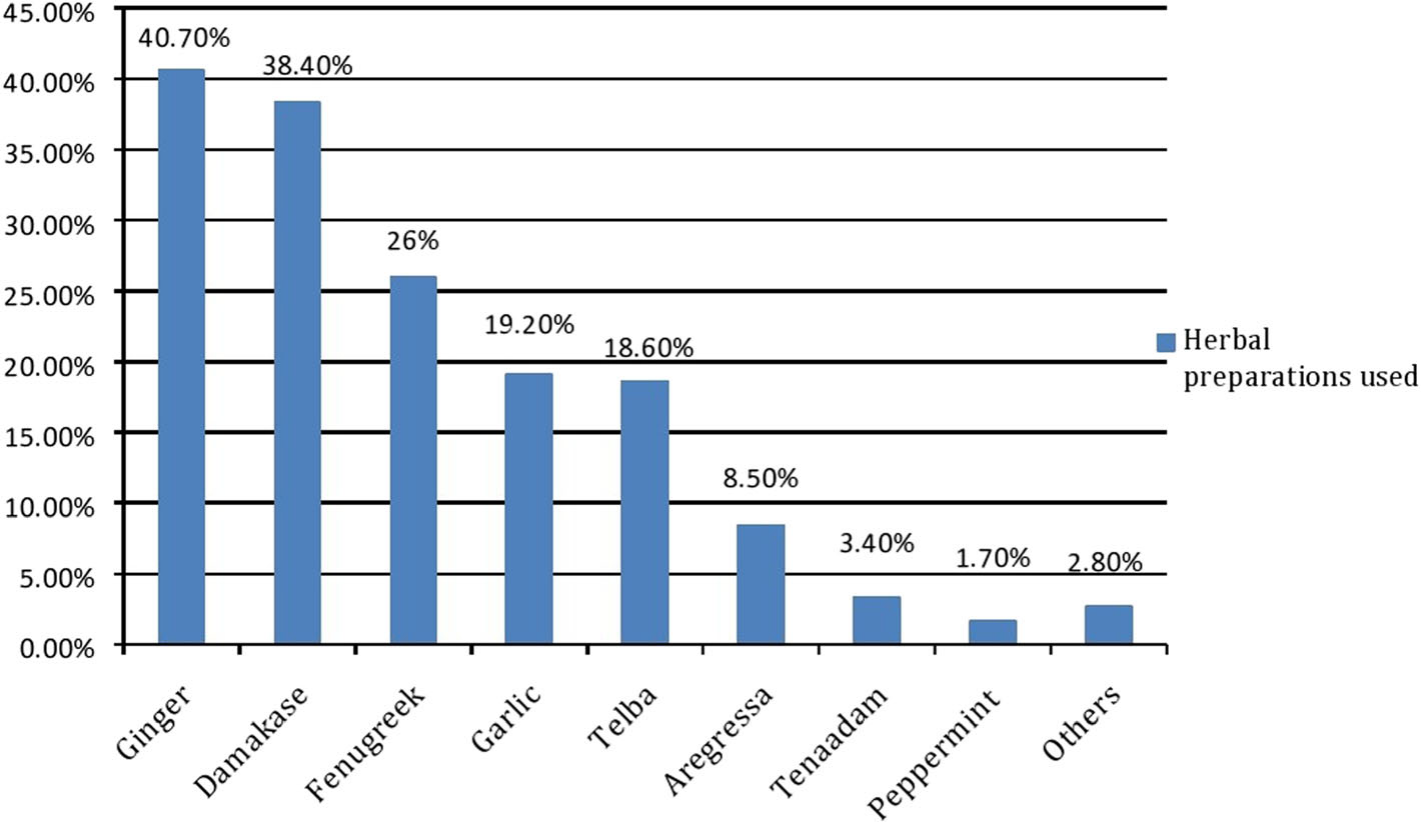
Herbal preparations used during current pregnancy, Gondar, 2016

**Fig. 2:**
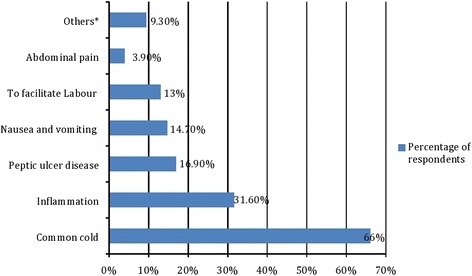
Indications for herbal medicine use during pregnancy, Gondar, 2016

Of the total respondents, 177 (48.6%) used herbal medicine during current pregnancy, with two third of them (68.4%) used during their third trimester. The most common herbal preparations used were ginger *(Zingiber officinale*) (40.7%) and damakasse (*Ocimum lamiifolium)* (38.4%) The most common indications for use were common cold (66%), and inflammation (31.6%). Herbal preparations used and their indications are summarized in Figs. 1 and 2.

Table 2 describes the characteristics of herbal medicine use among respondents. The most commonly cited source of information about herbal medicine was families, relatives and friends (46.9%) followed by other pregnant women who used herbal medicines (36.1%). The most commonly stated reason for using herbal medicine was “herbal medicines are cheap and accessible (54.8%)”, followed by “safe in pregnancy (18.6%)”. Similarly, the most common reason for not using herbal medicine among non-users was “lack of belief in the benefits of herbs (45.4%) followed by “afraid the side effect (31%)”. The majority of pregnant women who use herbal medicines (89.8%) did not discuss their use of herbal medicines with health care practitioners. Most of herbal medicine users (87.6%) reported that they haven’t experienced any apparent side effects from herbs. However, only31.1% of the respondents answered that they were satisfied with the result of herbal medicine use.

**Table 2:** Characteristics of herbal medicine use among respondents, Gondar, 2016 (*N* = 364)

Variables	Frequency, n (%)
Herbal medicine use during current pregnancy	
No	187 (51.4)
Yes	177 (48.6)
Source of information about herbal medicine^a^ (*n* = 177)	
Families, friends and relatives	83 (46.9)
Health care professionals	4 (2.2)
Media (internet, television, radio, book)	26 (14.7)
Pregnant women who used herbal medicines	64 (36.1)
Reasons for herbal medicine use^a^ (*n* = 177)	
Family, tradition or culture	22 (12.4)
Belief in effectiveness of herbal medicines	25 (14.2)
Herbal medicines are cheap and accessible	97 (54.8)
Treatment of other medical problems	13 (7.3)
Safe in pregnancy	33 (18.6)
Reason for not using herbal medicines among non-users (*n* = 187)	
Lack of belief in the benefits of herbs	85 (45.4)
Afraid the side effect	58 (31)
Lack of availability	8 (4.3)
Didn’t get sick during gestation	36 (19.2)
Discuss with HCPs about herbal medicine use (*n* = 177)	
No	159 (89.8)
Yes	18 (10.2)
Side effects from herbal medicine use (*n* = 177)	
No	155 (87.6)
Yes	22 (12.4)
Satisfaction with herbal medicine use (*n* = 177)	
Satisfied	55 (31.1)
Average	74 (41.8)
Dissatisfied	48 (27.1)

Abbreviation: *HCPs* Health care practitioners

^a^More than one option possible

### Factors associated with herbal medicine use

Variables that were significantly associated with herbal medicine use in the bivariate analysis were further examined in multivariate logistic regression. Accordingly, residence, educational status and average monthly income were found to have a significant association in multivariate logistic regression analysis. The odds of herbal medicine use during pregnancy was 3.15 times higher among rural residents as compared to urban residents (AOR: 3.15, 95% CI: 1.17–6.14). Pregnant women who were illiterate (no formal education) were 4.05 times more likely to use herbal medicine than those who attended tertiary education (AOR: 4.05CI: 2.48–6.62). It also showed that pregnant women who had average monthly income less than 100 USD were 3.08 times more likely to use herbal medicine than those who had average monthly income of greater than 200USD(AOR: 3.08CI: 1.22–7.77). There is no significant association between herbal medication use and age group, religion, education status, parity, previous ANC follow up or duration of pregnancy both in the bi-variate and multivariate logistic regression analysis (Table 1).

## Discussion

Currently, the use of herbal medicine is a common practice both in developed and developing country [16]. The present study aimed to assess the prevalence and correlates of herbal medicine use among pregnant women attending ANC clinic at University of Gondar referral and teaching hospital, northwest Ethiopia. According to the finding of our study, 48.6%used herbal medicine during current pregnancy, with two thirds (68.4%) reporting use during the third trimester. This finding is comparable with reports from western Ethiopia (50.4%) [14] and in Malaysia (51.4%) [16]. However, it was higher compared to findings from Kenya 12% [11], and Italy (27.8%) [17] and lower than a report from Southern Ethiopia [15]. The difference in prevalence may be due to variation in accessibility, affordability and cultural issues regarding herbal and modern medicine use between nations and districts of a country. The common practice of herbal medicine use in our study could be partially explained by the fact that the tradition and culture in Ethiopia encourages the use of herbal medicines, which is further augmented by the presence of many traditional medicine practitioners.

In our study, ginger (40.7%) is the most commonly used herbal medicines during pregnancy. Similarly, a study done in Alexandria revealed that ginger (52.4%) is the most commonly used herb in pregnant women [18]. However, in the study conducted in Virginia, peppermint (18%) was the most commonly used herb [19]. Other common herbal preparations reported in our study were fenugreek (26%), garlic (19.2%), telba (18.64%), aregressa (5.65%) and tenaadam (3.4%). The difference in pattern across different regions may be due to difference in accessibility and geographical distribution of herbs. However, the pattern of herbal medicine use in our study is almost similar to the study done in Hosanna town, southern Ethiopia where garlic, ginger, tenaadam and damakasse were reported to be the commonest herbs used by pregnant women [15]. Unlike the finding of this study, the common indications of herbal remedies were for labor facilitation (89.8%) followed by morning sickness (21%) in Malaysia [16] and for diarrhea (39.4%) and diabetes (31.6%) in Nigeria.

This indicates the presence of multiple claimed therapeutic roles of herbal preparations during gestational period which may need scientific clarification.

To prevent the possible harm imposed by the use of herbal medicines, health care providers should emphasize safety issues to pregnant women and make an effort to endow women with evidence-based information regarding herbal medicines. In our study, herbal medicine use was discussed with their health care providers by only 10.2% of pregnant women. The lack of communication between the health care providers and pregnant woman who are using herbal medicine may have a harmful effect on the mother as well as the fetus i.e. teratogenicity. Therefore, health care providers should acknowledge women’s’ use of herbal medicine, encouraging active conversation for the proper use of herbal medicines. The most common reasons cited for not using herbal medicines among non-users were lack of belief in the benefits of herbs (45.4%) followed by fear of side effects (31%). This finding corroborates the finding of the study conducted in Alexandria, where 44.5% of women do not trust in the benefits of herbs [18]. However, the Nigerian study reported that 33.4% of respondents trust in the benefits of herbs and 30.4% respondents believe that herbal medicines do not have adverse effects [6]. This may be due to socio-cultural difference among the respondents in study areas.

The odds of herbal medicine use during pregnancy were 3.15 times higher among rural residents as compared to urban residents. This may be due to difference in the accessibility of conventional medicine and health care center in rural and urban area. Pregnant women who were illiterate (no formal education) were 4.05 times more likely to use herbal medicine than those who attended tertiary education. This finding was in line with the study conducted in Hossana, Ethiopia, Nigeria [6, 20], Kenya [11], Nekemte [14] and Ghana [21]. It also showed that pregnant women who had average monthly income less than 100 USD were 3.1 times more likely to use herbal medicine than those who had average monthly income of greater than 200USD. This is similar with the study done in Malaysia [16]. This phenomenon might be explained by low cost of the traditional medicine compared to the modern medicine services [14].

### Limitations of the study

The study has some limitations that should be taken into account while interpreting the results. As the study is cross sectional and depends on self-reported assessment, under reporting is very likely. Thus, an exact cause and effect associations here in this study is difficult.

## Conclusion

The use of herbal medicine during pregnancy is a common practice and associated with rural residency, illiteracy and low average monthly income. Pregnant women depend mainly on family, friends and relatives as a source of information about herbal medicines. Commonly used herbs among pregnant women were ginger *(Zingiber officinale*) and damakasse (*Ocimum lamiifolium)* and the most common indication for use were common cold and inflammation. Given the high frequency of herbal medicine and a very low disclosure rate, health care providers should be open to discuss the use of herbal medicines with their pregnant women as it will lead to better health outcome. It is also recommended that commonly used herbal preparations should be further studied to certify their efficacy and safety.

## Additional file

Additional file 1: Questionnaire used for the study. (DOCX 28 kb)

## Abbreviations

ANC: Anti natal care
AOR: Adjusted odds ratio
CI: Confidence interval
OR: Odds ratio
SPSS: Statistical Package for the Social Sciences
UOGRTH: University of Gondar Referral and Teaching Hospital
USD: United States dollar
WHO: World Health Organization
