# Supplementary material for: Prevalence and associated factors of herbal medicine use among pregnant women on antenatal care follow-up at University of Gondar referral and teaching hospital, Ethiopia: a cross-sectional study
Source: BMC Complement Altern Med. 2017 Feb 1;17:86. doi: 10.1186/s12906-017-1608-4 (PMC5286838; doi:10.1186/s12906-017-1608-4)
Supplement: Additional file 1: — Questionnaire used for the study. (DOCX 28 kb) [file 12906_2017_1608_MOESM1_ESM.docx]

**University of Gondar**

**College of Medicine and Health Sciences, School of pharmacy**

**Consent Information sheet**

My name is __________________________. I am here on behalf of Gondar University research group staffs. We are conducting a research on **‘Use of herbal medicines among pregnant women attending in university of Gondar referral hospital, northwest Ethiopia: A cross sectional study.’** Their research project is approved by the Research Ethics committee of Gondar University. You are selected randomly to participate in this study. Your participation is purely based on your willingness. You have the right to choose not to take part in this study. If you choose to take part, you have the right to stop at any time. If you are willing to participate or refuse or decide to withdraw later, you will not be subjected to any ill-treatment.

If you agree to participate in the study, you will be asked to answer some questions about yourself and your family, your knowledge about herbal medicine use. The interview with you will take about 15 minutes. The study will explore about the use and correlates of herbal medicine use during pregnancy. It can also provide baseline data for policy makers and other researchers for further improvements of herbal medicine use in pregnancy. The information that you provide will be kept confidential by using only code numbers and locking the data.

Based on the understanding of the information I gave you, are you willing to participate in this study?

1. Yes 2. No

**Part I:** **Socio demographic and pregnancy related information of respondents**

| **Sr. No** | **Questions** | **Choice Answers** |
| --- | --- | --- |
| 101 | Sex | 1. Male 2. Female |
| 102 | Age | in years:__________ |
| 104 | Marital status | 1. Single (not married, separated, widowed, divorced) 2. Married/living with a partner |
| 105 | Religion | 1. Orthodox 2. Muslim 3. Protestant 4. Catholic 5. Other, specify__________ |
| 106 | Occupation status | 1. Employed 2. Unemployed |
| 107 | Educational Status | 1. Illiterate 2. Primary education(1-8) 3. Secondary education (9-12) 4. Tertiary education (college and above) |
| 108 | Average monthly family income | in USD___________ |
| 111 | Parity | 1. 1- 2 children 2. 3-4 children 3. >4 children |
| 112 | Do you have ANC visiting history before? | 1. Yes 2. No |
| 113 | Presence of health problem not related to gestation | 1. Yes 2. No |
| 114 | Trimester of current pregnancy | 1. 1^st^ Trimester 2. 2^nd^ Trimester 3. 3^rd^ Trimester |

**Part II: Herbal medicine use**

| 112 | Have you used herbal medicine during pregnancy? | 1. Yes, *if yes, when (month of use)? ________________* 2. No |
| --- | --- | --- |
| 113 | If you have not used herbal medicine, why not? | 1. Lack of belief in the benefits of herbs 2. Afraid the side effect 3. Lack of availability 4. Didn’t get sick during gestation |
| 115 | Why have you used herbal medicines? (more than one option is possible) | 1. Family, tradition or culture 2. Belief in effectiveness of herbal medicines 3. Herbal medicines are cheap and accessible 4. Treatment of other medical problems 5. Safe in pregnancy |
| 117 | What type of herbal medicines have you used? More than one answer is possible | 1. Ginger 2. Peppermint 3. Fenugreek 4. Damakesse 5. Garlic 6. Tenaadam 7. Telba 8. Others, please specify______________ |
| 118 | For what purpose do you use those herbal medications? | 1. Common cold 2. Inflammation 3. Nausea and vomiting 4. Abdominal colic 5. Peptic ulcer disease 6. Body swallowing 7. Headache 8. Others(specify)________ |
| 118 | What is your source of information about herbal medicines? | 1. Families, friends and relatives 2. Media (internet, television, radio, book) 3. Health practitioner 4. Pregnant women who used herbal medicines 5. Others, specify_______________ |
| 120 | Have you had any untoward effects from herbal medicines use? | 1. Yes 2. No |
| 121 | Have you ever discussed using herbal medicines with your Health care provider? | 1. Yes 2. No |
| 122 | In general, how could you rate the advantage you get from using herbal medicines use? | 1. Satisfied 2. Average 3. Dissatisfied |

**Thank you for your participation!!!**
